# Enhanced Protein Extraction from *Auxenochlorella protothecoides* Through Synergistic Mechanical Cell Disruption and Alkaline Solubilization

**DOI:** 10.3390/foods14152597

**Published:** 2025-07-24

**Authors:** Jun Wei Ng, Sze Ying Lee, Tong Mei Teh, Melanie Weingarten, Md. Mahabubur Rahman Talukder

**Affiliations:** Singapore Institute of Food and Biotechnology Innovation (SIFBI), Agency for Science, Technology, and Research (A*STAR), 31 Biopolis Way, Nanos, Singapore 138669, Singapore; ng_jun_wei@a-star.edu.sg (J.W.N.); lee_sze_ying@a-star.edu.sg (S.Y.L.); teh_tong_mei@a-star.edu.sg (T.M.T.); melanie_weingarten@a-star.edu.sg (M.W.)

**Keywords:** microalgae, wet biomass processing, mechanical cell disruption, high-pressure homogenization, alkaline solubilization, extraction

## Abstract

Microalgae proteins are increasingly recognized in the food and nutraceutical industries for their functional versatility and high nutritional value. Mild alkaline treatment is commonly used for cell wall degradation and intracellular protein solubilization, consequently enhancing the protein extraction yield. The findings of this study reveal that alkaline treatment alone, even at higher NaOH concentration (up to 0.3 M) and treatment time (up to 90 min), was ineffective (max. 2.4% yield) for the extraction of protein from *Auxenochlorella protothecoides* biomass. This challenge was significantly reduced through synergistic application of mechanical cell disruption using high-pressure homogenization (HPH) and alkaline solubilization. Single-pass HPH (35 k psi) alone without alkaline treatment led to 52.3% protein solubilization from wet biomass directly harvested from culture broth, while it was only 18.5% for spray-dried biomass. The combined effect of HPH and alkaline (0.1 M NaOH) treatment significantly increased protein extraction yield to 68.0% for a spray-dried biomass loading of 50 g L^−1^. Through replacing spray-dried biomass with wet biomass, the requirement of NaOH was reduced by 5-fold to 0.02 M to achieve a similar yield of 68.1%. The process integration of HPH with the mild alkaline solubilization and utilization of wet biomass from culture broth showed high potential for industrialization of microalgae protein extraction. This method achieves high extraction yield while reducing alkaline waste and eliminating the need for energy-consuming drying of biomass, thereby minimizing the environmental impact.

## 1. Introduction

Single-cell protein (SCP) or protein-rich microbial biomass is increasingly important due to its sustainability, nutritional value, and potential applications in food, feed, and biotechnology. While yeast/fungus and bacteria remain valuable SCP sources due to their fast growth, microalgae offer superior sustainability, high protein content (50–70%) [1], broader nutritional diversity including bioactive, and a lower environmental impact. Additionally, they utilize sunlight and CO_2_, making them a highly attractive alternative for future protein production. However, the rigid cell wall of microalgae like *Chlorella vulgaris* and limited solubility of microalgal protein in water pose a significant challenge, leading to low intracellular component availability and hindering efficient protein extraction [2]. Hence, an effective method for cell disruption and intracellular protein solubilization is a prerequisite to preparing microalgae protein isolate/concentrate. Furthermore, such a method should not only maximize protein recovery yield but must be scalable, cost-effective, and avoid degradation of the protein. The protein degradation or hydrolysis affects the protein attributes (e.g., increases bitter taste and alters protein functionalities). Elevated NaOH concentrations (pH > 12) can lead to protein hydrolysis, reducing functional properties [3].

A chemical approach using very mild alkaline water (pH up to 9.5) is commonly used in the industrial extraction of plant proteins, such as soy protein isolates or concentrates. This process permeabilizes plant cell walls, solubilizes proteins, and enables subsequent recovery via isoelectric precipitation and drying. However, our recent findings suggest that the mild alkaline treatment (pH 9.0) is ineffective in permeabilizing the rigid cell wall of C. vulgaris, resulting in limited protein solubilization (yield 6%) at a biomass load of 50 g L^−1^ [4]. Safi et al. [5] also reported a modest protein recovery of 26% from *C. vulgaris* using alkaline treatment at an elevated pH of 12 for 24 h for a biomass load of 20 g L^−1^. The authors found that the sodium hydroxide solution was insufficient to completely hydrolyze the cell wall of *C. vulgaris* but partially weakened it, allowing the release of only small-sized cytoplasmic proteins while hindering the diffusion of larger proteins. We also observed that increasing NaOH concentration enhances protein solubilization; however, a higher NaOH level (>0.1 M) leads to degradation of *Chlorella* protein [4]. Additionally, higher NaOH levels contribute to increased sodium content in the final product, which may be undesirable for food applications.

Numerous studies on physical cell disruption using bead milling, HPH, or ultrasonication have been reported for enhancing microbial cell permeabilization, resulting in higher protein extraction. A study by Postma et al. [6] demonstrated that bead milling resulted in more than 97% cell disintegration, yielding up to 42% of water-soluble proteins from *C. vulgaris*. Safi et al. [5] reported that HPH rapidly disrupted the cells, allowing the recovery of 66% of total proteins from *C. vulgaris*. Jubeau et al. [7] optimized the HPH process operating pressure and number of passages for the selective extraction of proteins and pigments from *Porphyridium cruentum*. Perez et al. [8] enhanced microalgae protein extraction using emerging cell disruption technology, pulsed electric field (PEF). Although PEF could enhance the extraction yield from 11 to 25%, the improved yield is not that high and is lower than HPH. Katsimichas et al. [9] studied the kinetics of HPH-assisted protein extraction and reported protein recovery of 38% after 24 h through aqueous extraction. While previous studies on HPH and bead milling showed promising results for microalgae cell disruption, they did not compare the efficacy of these two methods in the context of both cell disruption and subsequent alkaline protein solubilization. More importantly, these studies did not focus on fine-tuning the concentration of alkaline, a critical factor that not only influences protein solubilization but can also lead to protein degradation and elevated waste generation. Furthermore, the prior work primarily used dried microalgae biomass. The impact of HPH and bead milling on protein solubilization using wet biomass harvested directly from fermentation broth remains unexplored. The utilization of wet biomass eliminates not only the energy-intensive drying step but also simplifies the overall microalgal biomass production process.

This study aims to develop a physico-chemical protein extraction process from wet microalgae biomass to avoid energy-consuming drying and reduce waste generation, an unfulfilled need to prepare microbial protein isolate/concentrate at an industrial scale. Our primary focus was on developing and optimizing a scalable extraction method, and we prioritized yield as initial performance indicator. The reduction of alkaline loading, which affects the protein quality, is another objective of this study. Heterotrophically, lab-grown *A. protothecoides* biomass obtained from cultured broth in a bioreactor was used to optimize HPH and bead milling to compare their effectiveness. Alkaline concentration was fine-tuned to maximize protein solubilization and extraction yield. The spray-dried *Auxenochlorella* biomass served as a control to evaluate the benefits of utilizing wet biomass in terms of protein extraction yield, reduced process severity, and lower alkaline concentration. *A. protothecoides*, formerly *Chlorella protothecoides*, is a versatile microalga capable of autotrophic, mixotrophic, and heterotrophic growth. With a protein content of approximately 50%, it is a promising sustainable protein source. However, research on its protein extraction methods remains limited compared to other microalgae strains like *C. vulgaris*.

## 2. Materials and Methods

### 2.1. Chemicals and Reagents

Sodium hydroxide pellet, yeast extract, glucose, Bradford reagent, and bovine serum albumin (BSA) were supplied by Sigma-Aldrich (St. Louis, MO, USA), while all other chemicals used in the study were of analytical grade. All the materials were used without further purification. The water used throughout the work was ultra-pure water treated by a Milli-Q integral water purification system.

### 2.2. Microalgae Cultivation

The microalgae selected was *A. protothecoides* SAG 211-7a, obtained from the University of Göttingen. A modified Bold’s Basal Medium (BBM) in which 6.7 g L^−1^ of yeast extract replaced NaNO_3_ was prepared for microalgae cultivation, with the composition (g L^−1^): yeast extract, 6.7; CaCl_2_.2H_2_O, 0.025; MgSO_4_.7H_2_O, 0.075; K_2_HPO_4_, 0.075; KH_2_PO_4_, 0.175; NaCl, 0.025; H_3_BO_3_, 0.114; ethylenediaminetetraacetic acid, 0.05; KOH, 0.031; FeSO_4_.7H_2_O, 0.00498; ZnSO_4_.7H_2_O, 0.0088; MnCl_2_.4H_2_O, 0.0014; MoO_3_, 0.0007; CuSO_4_.5H_2_O, 0.0016; and Co(NO_3_)_2_.6H_2_O, 0.0005. For liquid precultures, microalgae were incubated in the modified BBM with 20 g L^−1^ glucose for 30 h in a shake flask under heterotrophic conditions (200 rpm, 25 °C, in darkness). These precultures were used to inoculate the 10 L bioreactor cultures with the modified BBM containing 48 g L^−1^ glucose and 40 g L^−1^ yeast extract. The cultivation was performed under batch mode in a Biostat B-DCU 10 L bioreactor (Sartorius, Göttingen, Germany) with a 10% (*v*/*v*) inoculum from the pre-culture. The cultivation conditions applied were a temperature of 25 °C, pH 6.5, and dissolved oxygen maintained at 50% with a cascade system under dark conditions. The cultivation was completed when the residual glucose was depleted.

### 2.3. Protein Extraction from A. protothecoides

#### 2.3.1. Preparation of Microalgae Biomass

Biomass was harvested at the stationary growth phase of the microalgae. The broth was centrifuged at 3000× *g* for 10 min using a Sorvall Lynx 4000 centrifuge (Thermo Fisher Scientific, Waltham, MA, USA) to remove the liquid content. The biomass pellet obtained was either used directly or spray-dried for protein extraction. The spray-dried conditions were inlet temperature 190 °C, outlet temperature 100–105 °C, feed pump rate 12 mL min^−1^, and air blower 30 Hz using YC-015 Nano Spray Dryer (Pilotech, Shanghai, China). The protein content of *A. protothecoides* biomass was determined using the Dumas combustion method in a DUMATHERM N Pro analyzer (Gerhardt Analytical Systems, Königswinter, Germany). A nitrogen-to-protein conversion factor of 6.25 was used [10], and the protein content of both wet and spray-dried biomass was 43% on a dry basis.

#### 2.3.2. Protein Extraction Using Alkaline Extraction Alone

Alkaline extraction was applied to wet and spray-dried biomass to obtain microalgae protein. The biomass (5% dry matter) was mixed with 5 mL of sodium hydroxide (NaOH) while stirring at 400 rpm and 37 °C. Different NaOH concentrations (0 to 0.3 M) and mixing times (5 to 90 min) were tested to evaluate their effects on the solubilization yield of the microalgae protein. After mixing, the solution was centrifuged at 3000× *g* for 10 min, and the protein content of the supernatant was determined using the Bradford assay, as stated in Section 2.4. The solubilization yield (%) was calculated using Equation (1):

(1) $$\mathrm{Protein}\;\mathrm{solubilization}\;\mathrm{yield}\;\left(\%\right)\;=\;\frac{\mathrm{Solubilized}\;\mathrm{protein}\;\mathrm{in}\;\mathrm{supernatant}}{\mathrm{Total}\;\mathrm{protein}\;\mathrm{in}\;\mathrm{biomass}}\;\times \;100$$

#### 2.3.3. Protein Extraction Using High-Pressure Homogenization Followed by Alkaline Treatment

The wet and spray-dried biomass concentrations were adjusted to 5% dry matter (50 g L^−1^) and subjected to HPH in a CF2 cell disruptor (Constant Systems, Daventry, United Kingdom), followed by alkaline solubilization with NaOH. Initially, the biomass was homogenized at 4 °C up to four passes at 35 k psi, with a constant flow rate of 405 mL min^−1^. After each pass, samples were collected and centrifuged. The soluble protein content in the supernatant was then measured using the Bradford method (Section 2.4) to evaluate the protein solubilization yield using Equation (1).

Subsequently, the homogenized samples were treated with varying NaOH concentrations (0.00002 to 0.3 M) at 37 °C and stirred at 400 rpm for durations ranging from 5 to 60 min. The mixtures were centrifuged to obtain the supernatant, and their protein solubilization yield was determined using Equation (1).

#### 2.3.4. Protein Extraction Using Bead Milling Followed by Alkaline Treatment

The concentration of both wet and spray-dried biomasses was adjusted to 5% dry matter (50 g L^−1^) in the water. For each treatment, 20 mL of the biomass suspension (5% dry matter) and 20 mL of glass beads were transferred into a 50 mL Falcon tube. The samples were then subjected to bead milling using a Retsch Mixer Mill MM 400 (Retsch, Haan, Germany) under the following conditions: frequency of 26 Hz for 6 cycles of 10 min each, with a 5-min break between cycles. This milling frequency was selected based on our previous findings, which showed that 26 Hz was the most effective in enhancing protein solubilization from dried *C. vulgaris* biomass [11]. The same condition was applied here to assess its effectiveness in disrupting *A. protothecoides* cells and releasing intracellular proteins under varying alkaline conditions.

Following milling, the glass beads were separated, and the resulting biomass slurry was collected. NaOH was added to the biomass slurry to achieve final concentrations ranging from 0.005 to 0.2 M. To solubilize the protein, the mixture was stirred at 400 rpm and 37 °C for 5 min and centrifuged. The protein content in the supernatant before and after alkaline treatment was analyzed using the Bradford assay (Section 2.4), and solubilization yield was estimated based on Equation (1).

### 2.4. Determination of Soluble Protein Content

The soluble proteins in the supernatant were quantified using the Bradford method [12], as used by other researchers for microalgae protein [13]. A total of 50 µL of sample was mixed with 1.5 mL of Bradford reagent and measured at the wavelength of 595 nm. Protein was quantified using a calibration curve prepared using BSA as a standard.

### 2.5. Statistics

Each experiment was performed in triplicate under consistent conditions, and all data are expressed as mean ± standard deviation. Error bars in figures represent the standard deviation of mean values. Statistical analysis was conducted using analysis of variance (ANOVA) and Tukey’s multiple comparisons in SPSS software version 28.0 (IBM, Armonk, NY, USA) to determine significant differences between mean values at a 95% confidence level (*p* < 0.05).

## 3. Results and Discussion

### 3.1. Alkaline Solubilization of Protein

#### 3.1.1. Effect of NaOH Concentration on Protein Solubilization Yield

**Figure 1:**
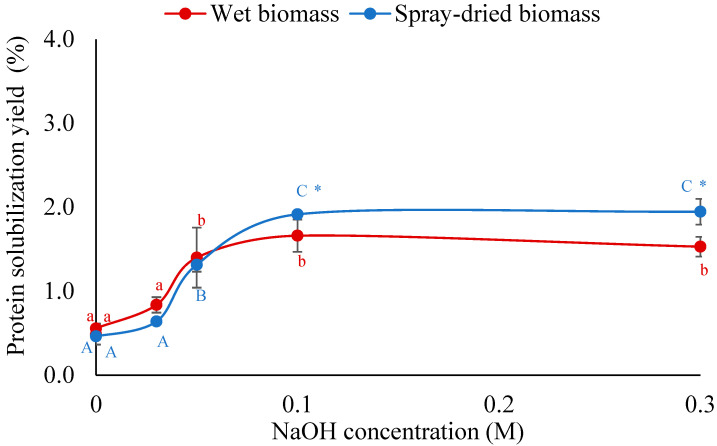
Effect of NaOH concentration on protein solubilization of wet and spray-dried *A. protothecoides* biomass during alkaline treatment. Different lowercase and uppercase letters indicate significant differences between treatments at different NaOH concentrations for wet biomass and spray-dried biomass, respectively (*p* < 0.05). Asterisks (*) indicate significant differences between biomass types (wet vs. spray-dried) at the same NaOH concentration (*p* < 0.05).

Both wet and spray-dried *Auxenochlorella* biomass were treated with different alkaline (NaOH) concentrations at a temperature of 37 °C and mixing at 400 rpm for 1 h to understand the effectiveness of breaking the rigid cell wall of *A. protothecoides* and solubilization of intracellular protein through disruption of the complex protein–polysaccharide–lipid structure and deprotonation of acidic amino acids. The results are shown in Figure 1. It can be seen that the protein solubilization yield slightly increased with the increase in NaOH concentrations for both wet and spray-dried biomass, but the solubilization yield was very low (~2.0%), even at elevated concentrations of 0.3 M. It should be noted that after NaOH concentration of 0.1 M, the protein solubilization yield remained the same with the increase in concentration. This result suggests that alkaline treatment alone is not sufficient for breaking the rigid, highly cross-linked cell walls composed of polysaccharides, phospholipid, and glycoprotein [14].

#### 3.1.2. Mixing Time for Protein Solubilization

To further investigate the efficacy of alkaline treatment alone on cell breaking and protein solubilization, the mixing time was varied while NaOH concentration was kept constant at 0.1 M NaOH. The NaOH concentration of 0.1 M was chosen based on the result demonstrated in Figure 1. It can be seen from Table 1 that extending the mixing time from 60 to 90 min resulted in only a slight improvement in protein yield, increasing from 1.66% to 2.436% for wet biomass and from 1.915% to 2.274% for spray-dried biomass. Despite this improvement, the overall yields remained low and insignificant, further supporting the conclusion that alkaline treatment alone is insufficient to effectively solubilize microalgal proteins. To enhance protein solubilization, microalgae biomass was physically disrupted using HPH and bead milling. The disrupted biomass was then subjected to alkaline treatment with varying NaOH concentration, as detailed in the following sections.

### 3.2. High-Pressure Homogenization for Enhanced Solubilization of Protein

#### 3.2.1. Effect of Number of HPH Passes

**Figure 2:**
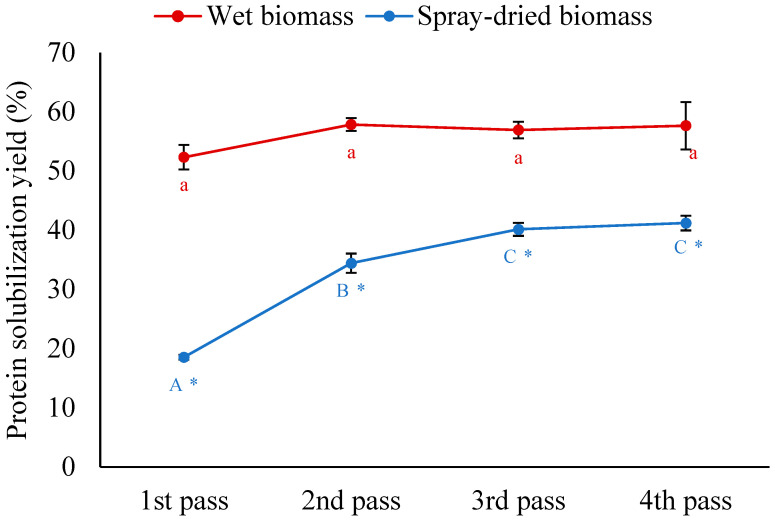
Effect of pass number on solubilization of wet and spray-dried *A. protothecoides* biomass during HPH with water. Different lowercase and uppercase letters indicate significant differences between treatments at different HPH passes for wet biomass and spray-dried biomass, respectively (*p* < 0.05). Asterisks (*) indicate significant differences between biomass types (wet vs. spray-dried) at the same HPH pass (*p* < 0.05).

HPH can disrupt cells through high shear forces, turbulence, and cavitation to break microbial cells. The efficiency of cell disruption and the release of intracellular components depends on both the applied pressure and the number of HPH passes. In this study, the concentration of both the wet and spray-dried biomasses was adjusted to 50 g L^−1^ (5% dry matter) using water, and the effect of the number of HPH passes was investigated at an operating pressure of 35 k psi with the temperature maintained at 4 °C. It is evident from Figure 2 that the overall yields for both biomass types treated with HPH were significantly improved compared to alkaline extraction alone (Figure 1), thus highlighting the effectiveness of mechanical disruption in breaking the biomass to facilitate protein release. For the wet biomass, the solubilization yield plateaued at approximately 58%, with no significant differences observed between the first and subsequent passes. This indicates that the cell disruption of the wet biomass was highly effective after a single pass. On the other hand, spray-dried biomass exhibited a progressive increase in solubilization yield with the number of passes, rising from 18.5% after the first pass to around 41% after the fourth pass.

The lower yields for spray-dried biomass using HPH, compared to wet biomass, were likely caused by structural changes induced by high-pressure atomization and high temperature during the spray drying process [15,16]. The exposure to the high spray drying temperature of 190 °C might lead to protein denaturation and aggregation, reinforcing the cell envelope and reducing the protein solubility in water. Additionally, the lipid could undergo phase transition, reducing cell wall permeability. Some structural changes, such as membrane fluidity, can partially recover through hydration with water; however, protein denaturation and lipid phase transition may not be fully reversed. Hence, protein solubilization yield from the spray-dried biomass, even after four passes (41.2%), was lower than that achieved with wet biomass after the first pass (52.3%). Further studies are required to better understand the cellular changes induced by the spray drying process, which would improve the biorefinery process development. Since the difference in wet biomass protein solubilization yield between the first and second passes of HPH was only about 10% but required twice the energy, the single-pass HPH was chosen for subsequent process optimization.

#### 3.2.2. HPH Followed by Alkaline Treatment for Enhanced Protein Solubilization

**Figure 3:**
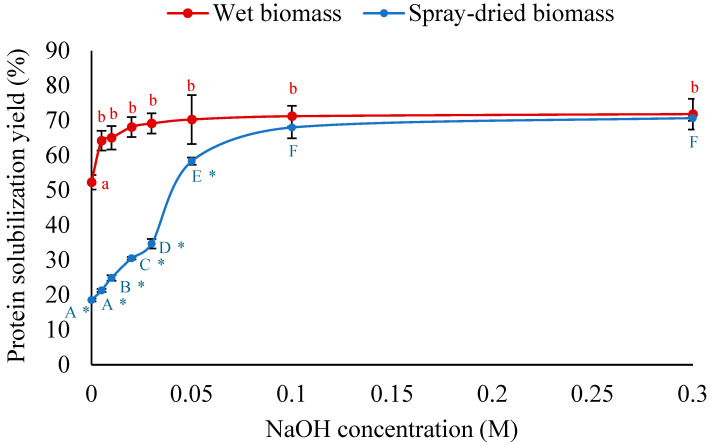
Effect of alkaline concentration on solubilization of first-pass homogenized wet and spray-dried *A. protothecoides* biomass. Different lowercase and uppercase letters indicate significant differences between treatments at different NaOH concentrations for wet biomass and spray-dried biomass, respectively (*p* < 0.05). Asterisks (*) indicate significant differences between biomass types (wet vs. spray-dried) at the same NaOH concentration (*p* < 0.05).

Following the first pass HPH with water, NaOH was added to adjust different alkaline concentrations, and the sample was mixed for 60 min for enhanced protein solubilization. An increasing trend in protein solubilization yield was observed for both wet and dry biomasses with the increase in NaOH concentration (Figure 3). The solubilization yield for dried biomass reached 68.0% at 0.1 M NaOH and remained almost the same at higher concentrations. In contrast, a similar yield of 68.1% was achieved with wet biomass at a 5-fold lower NaOH concentration of 0.02 M. Further increasing the NaOH concentration to 0.1 M led to a slight increase in yield to 70.3%. Notably, without NaOH treatment, about 2.8-fold more protein was released from the wet biomass (52.3%) compared to the dried biomass (18.5%) under the same conditions. These results suggest that the extraction of protein from wet biomass is more feasible in terms of enhanced solubilization yield, reduced energy consumption, and lower waste generation.

**Figure 4:**
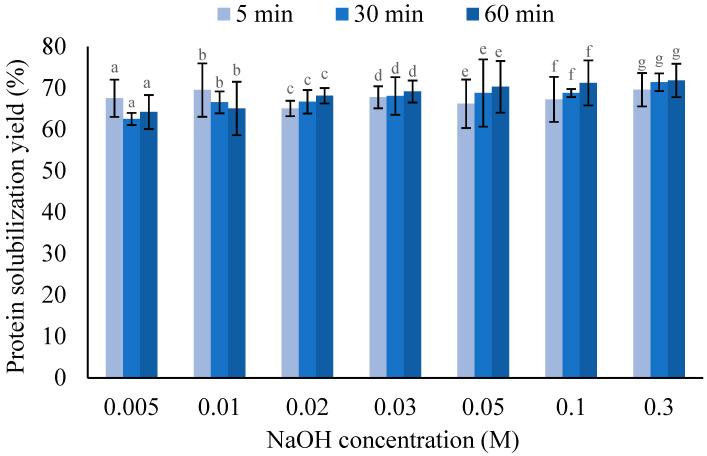
Effect of mixing time on protein solubilization yield of first-pass homogenized wet and spray-dried *A. protothecoides* biomass during alkaline solubilization step under different NaOH concentrations. Different lowercase letters indicate significant differences between treatments at different mixing times of the same NaOH concentration (*p* < 0.05). Asterisks (*) indicate significant differences between NaOH concentrations at the same mixing time (*p* < 0.05).

Figure 4 shows the impact of mixing time on the alkaline solubilization of HPH-treated wet biomass across different NaOH concentrations. It can be seen that a shorter mixing time of 5 min is sufficient for the solubilization of protein, and no considerable changes were observed with a longer mixing time. This result suggests that *A. protothecoides*’ proteins after HPH-induced cell disruption could instantly be soluble in alkaline water, and the protein solubilization after HPH was mainly driven by alkaline concentration.

### 3.3. Effect of Bead Milling on Protein Solubilization

**Figure 5:**
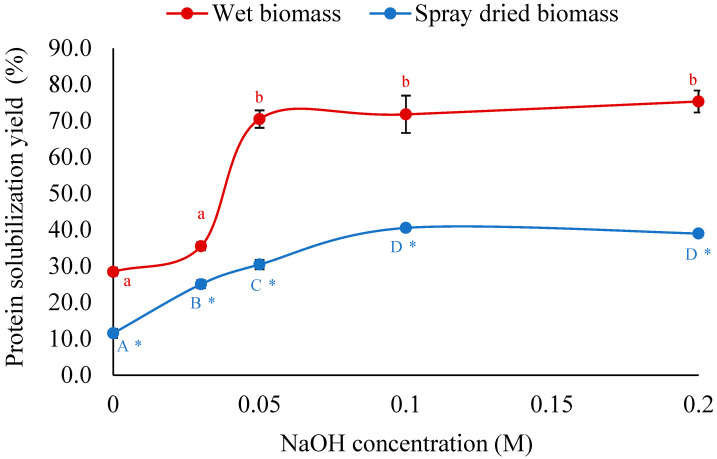
Effect of NaOH concentration on protein solubilization of bead-milled wet and spray-dried *A. protothecoides* biomass during alkaline solubilization step. Different lowercase and uppercase letters indicate significant differences between treatments at different NaOH concentrations for wet biomass and spray-dried biomass, respectively (*p* < 0.05). Asterisks (*) indicate significant differences between biomass types (wet vs. spray-dried) at the same NaOH concentration (*p* < 0.05).

Because HPH induces cell rupture through a combination of sudden pressure drop, shear force, turbulence, and cavitation, it is usually preferred for soft- to moderate-walled cells. In contrast, bead milling, where agitation causes the collision between beads and cells to physically break them, may be more effective for tough-walled cells. Hence, bead milling followed by alkaline treatment was applied to wet and spray-dried *Auxenochlorella* biomass for enhanced protein extraction. It is evident from Figure 5 that protein solubilization yields for both bead-milled wet and spray-dried biomasses increased with increasing NaOH concentrations. The maximum solubilization yield with wet biomass reached 70.5% at 0.05 M NaOH, about 2.5-fold higher than that achieved with dried biomass under the same conditions. This result further confirms that proteins from wet biomass are readily released into alkaline solution compared to those in spray-dried biomass, and that cell disruption is essential prior to alkaline treatment for enhanced protein extraction.

### 3.4. Comparison Between HPH and Bead Milling for Protein Solubilization

**Figure 6:**
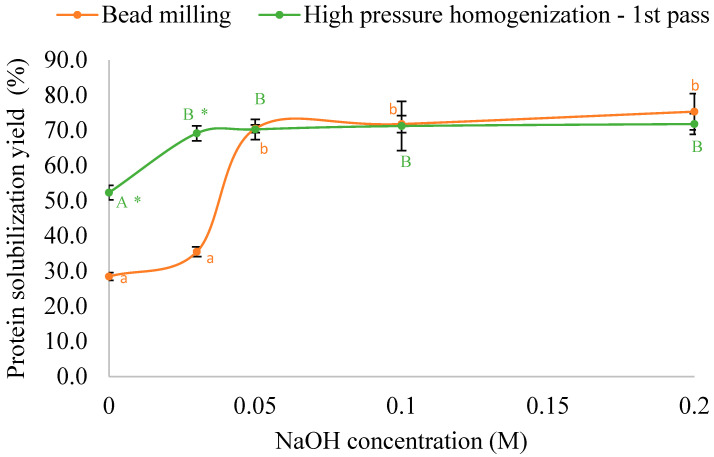
Comparison of protein solubilization yield between bead-milled and first-pass homogenized wet *A. protothecoides* biomass during the alkaline solubilization step. Different lowercase and uppercase letters indicate significant differences between treatments at different NaOH concentrations for the bead-milling method and high-pressure homogenization method, respectively (*p* < 0.05). Asterisks (*) indicate significant differences between the bead milling and high-pressure homogenization methods at the same NaOH concentration (*p* < 0.05).

The effectiveness of HPH (35 k psi, single pass) and bead milling (26 Hz, 60 min) was compared in the solubilization of protein from wet biomass at different NaOH concentrations (Figure 6). Both pre-treatment methods improved protein solubilization. For bead-milled biomass, alkaline treatment had minimal impact at a lower NaOH (<0.03 M) concentration, while a sharp increase in solubilization yield was observed between 0.03 and 0.05 M NaOH. The maximum yield reached 70.5% after which it plateaued. In contrast, the HPH process yielded a comparable yield at a lower NaOH concentration of 0.03 M, demonstrating superior efficacy over bead milling. It is worth noting that the HPH process yielded 1.8-fold higher protein solubilization (52.3%) without any NaOH (at 0.0 M) compared to bead milling (28.5%). These results suggest that HPH is preferred to disrupt *A. protothecoides* microalgal cells for enhanced protein extraction.

Alternative cell disruption methods have also been utilized to extract protein from *A. protothecoides*. Perez et al. [8] reported improved protein extraction yields by combining PEF or ultrasonication (US) with a 24 h incubation after treatment, resulting in a 122% increase in yield for PEF (from 11% to 25%) and 51.9% for US (from 13% to 19%). While PEF and US could be more energy efficient compared to HPH, the longer incubation time of 24 h makes the overall process slower. However, the proposed HPH method shows an advantage of being more effective, yielding higher protein recovery up to 70% without the need for extended incubation, which provides a more time-efficient and potentially more scalable solution for protein extraction from wet microalgal biomass. Further investigation is necessary to evaluate the energy efficiency of the proposed method. Additionally, a thorough assessment of protein quality and functional properties remains important regardless of PEF, US, and HPH. Mechanical cell disruption methods have been shown to significantly enhance protein digestibility without markedly altering amino acid profiles [17]. Moreover, the effects of cell disruption on sensory characteristics should be examined, as they can greatly impact the flavor profile and consumer acceptability of microalgae-based food products. For example, it was reported that HPH processing of *Nannochloropsis* generates intense grassy and fishy odors caused by elevated levels of fatty acid-derived unsaturated aldehydes, ketones, and alcohols [18].

## 4. Conclusions

A method that eliminates energy-consuming microalgae biomass drying and significantly reduces the requirement of NaOH concentration was developed for effective extraction of microalgae protein. HPH alone without alkaline treatment released 52.3% of protein from wet microalgae biomass, which is 2.8-fold higher compared to that achieved using spray-dried biomass (18.5%). HPH with subsequent mild alkaline treatment further increased the protein extraction yield from 52.3 to 68.1% with 0.02 M NaOH. The protein extraction yield further increased to 71.2% using a higher NaOH concentration. However, high NaOH concentrations could adversely affect the protein attributes for food applications. The reduced alkaline loading is particularly important in large-scale production, where the environmental impact of chemicals and waste disposal is a crucial consideration. Furthermore, it could potentially contribute to higher-quality protein extracts with more promising techno-functional properties.

Additionally, HPH proved superior to bead milling for disrupting microalgae cell walls, enabling protein extraction at a reduced NaOH concentration. The maximum protein extraction yield with bead milling was 70.5% using 0.05 M NaOH, whereas HPH achieved the same yield with only 0.03 M NaOH. Notably, HPH alone without alkaline treatment led to a 1.8-fold protein solubilization compared to bead milling.

Future research should focus on investigating the techno-functional properties, including the quality, such as the amino acid composition, digestibility, and bioactivity of the protein extracts obtained using this optimized method. These properties are critical for the use of microalgae proteins in food and nutraceutical applications.

## Figures and Tables

**Table 1:** Effect of mixing time on solubilization of wet and spray-dried *A. protothecoides* biomass during alkaline treatment with 0.1 M NaOH. Different lowercase and uppercase letters indicate significant differences between treatments at different mixing times for wet biomass and spray-dried biomass, respectively (*p* < 0.05). Asterisks (*) indicate significant differences between biomass types (wet vs. spray-dried) at the same mixing time (*p* < 0.05).

Mixing Time (min)	Protein Solubilization Yield (%)	
	Wet Biomass	Spray-Dried Biomass
5	0.454 ± 0.005 ^a^	1.078 ± 0.055 ^A *^
10	0.488 ± 0.035 ^a^	1.419 ± 0.152 ^B *^
20	0.835 ± 0.056 ^b^	1.621 ± 0.087 ^B *^
30	1.043 ± 0.039 ^c^	1.662 ± 0.129 ^C *^
40	1.534 ± 0.079 ^d^	1.857 ± 0.038 ^CD *^
50	1.600 ± 0.086 ^d^	1.872 ± 0.145 ^D *^
60	1.881 ± 0.144 ^e^	1.911 ± 0.041 ^D^
90	2.436 ± 0.060 ^f^	2.274 ± 0.021 ^E *^

## Data Availability

The original contributions presented in the study are included in the article, further inquiries can be directed to the corresponding author.
